# Win-stay/lose-switch, prospecting-based settlement strategy may not be adaptive under rapid environmental change

**DOI:** 10.1038/s41598-020-79942-3

**Published:** 2021-01-12

**Authors:** Janusz Kloskowski

**Affiliations:** Institute of Zoology, Poznań University of Life Sciences, ul. Wojska Polskiego 71C, 60-625, Poznań, Poland

**Keywords:** Ecology, Evolution

## Abstract

Understanding animal responses to environmental change is crucial for management of ecological traps. Between-year habitat selection was investigated in red‐necked grebes (*Podiceps grisegena*) breeding on semi-natural fish ponds, where differential stocking of fish created contrasting yet poorly predictable brood-stage food availabilities. Grebes lured to low-quality ponds were more likely to shift territories than birds nesting on high-quality ponds, and tended to move to ponds whose habitat quality had been high in the previous year, irrespective of the current quality of the new and old territories. The territory switchers typically visited their future breeding ponds during or immediately after the brood-rearing period. However, owing to rotation of fish stocks, the habitat quality of many ponds changed in the following year, and then switchers from low-quality ponds and stayers on previously high-quality ponds were ecologically trapped. Thus, although breeders were making an informed choice, their settlement decisions, based on the win–stay/lose–switch rule and prospecting a year in advance, were inappropriate in conditions of year-to-year habitat fluctuations. Effective adaptation to rapid environmental change may necessitate both learning to correctly evaluate uncertain environmental cues and abandonment of previously adaptive decision-making algorithms (here prioritizing past-year information and assuming temporal autocorrelation of habitat quality).

## Introduction

Habitat selection is essentially related to individual fitness, and therefore strong selective pressures should lead individuals to exploit environmental information and make optimal settlement decisions^1–3^. However, when put in novel environments in which they have not evolved, animals of many species have been documented to prefer habitats that do not maximize their fitness. Inattention to or misinterpretation of cues on habitat quality associated with human-induced rapid environmental change (HIREC) may result in ecological traps that drive populations toward decline^4–6^. Conceptual frameworks aiming to address HIREC posit that animal cue-response systems, ‘Darwinian algorithms’^4,7^, i.e. cognitive programs that structure and integrate experience and memory into meaningful frameworks, were shaped by selection pressures in prior environments and may underlie inappropriate behavioural responses in novel conditions^8,9^. Increasing attention has recently been paid to means of behavioural readjustment as an escape from ecological traps associated with HIREC, either via natural selection of genetically determined traits or via learning^8–10^. Learning may be of vital importance for the fate of populations, because natural selection processes are often unlikely to keep pace with HIREC^11,12^. Some types of traps leave no chance for learning because they provide only a single opportunity to respond during the animal's life cycle. However, provided the traps are not lethal, long-lived animals that have initially responded sub-optimally to habitat traps have the chance to modify their behaviour to avoid detrimental options^12,13^. On the other hand, learning may not always help to avoid traps^14^; evolved biases can prevent animals from behaving optimally in complex situations^15^. Empirical studies on whether animals that have been trapped in unpredictable habitats of inferior quality are capable of using the acquired experience to choose a better breeding situation in the subsequent reproductive cycle are lacking. A generally accepted simple decision algorithm that combines information about past breeding success and habitat options is that experienced individuals should display site fidelity if the current breeding site has yielded fitness benefits (‘win-stay’); conversely, they should attempt to improve their breeding conditions in the next reproductive cycle by shifting to other sites if the fitness outcomes were poor (‘lose-switch’)^3,16–18^ (but see^19,20^). To reduce environmental uncertainty, animals intending to move away should gather information on potential future territories^21^. Since the acquired information cannot be perfect, perception of habitat cues and sampling strategies used to prospect for potential breeding sites can be decisive for the learning outcomes^2,22^. The adaptiveness of the information used is constrained by its predictability over space and time^2,3^. Also, the individual fitness and population consequences of the use of misleading information by animals encountering ecological traps may differ from the consequences of uncertain/limited information or a random choice strategy^23^.

Here, decision rules adopted for between-year habitat selection were investigated in red-necked grebes (*Podiceps grisegena*) nesting on fish ponds, where food resources for breeding birds were determined by the density and size structure of the stocked fish. Red-necked grebes (hereafter ‘grebes’) are migrants; the natural breeding habitats of the European subspecies *grisegena* are shallow, eutrophic waters, such as shallow lakes, ponds and lagoons, with abundant emergent and submerged vegetation ensuring a rich supply of macroinvertebrate prey. In much of central and eastern Europe these habitats have been largely modified or destroyed due to fish introductions, climatic changes or wetland conversion. Presently, most of the population breeds on open ponds used to culture cyprinid fishes^24^. As generalist foragers, grebes are involved in strong interactions with fish, which may form their major food supply. However, many fish species grow to a refuge size from the gape‐limited predator and are capable of strong competition-like effects, i.e. eliminating aquatic insects and amphibians, the main alternative prey of grebes. This non-fish prey may be vitally important for the survival of hatchlings that cannot ingest large prey during the critical early period^25^. In the study area, grebes nearly exclusively used—showing little or no preference—two habitats differing in availability and dynamics of prey. These were ponds with low food abundance (low densities of small-sized fish) in the pre-laying season, but increasing to high abundance during the chick stage, and ponds containing fish attractive as food for settling birds but of unsuitable size for chicks (an ecological trap habitat resulting in heavy brood losses)^26,27^. Fish pond aquaculture offered a good opportunity to study behavioural responses to a HIREC trap. Differential fish stocking created a clear dichotomy in habitat quality among well-defined habitat patches; the suboptimal habitat provided alluring cues, while the habitat quality of individual patches (ponds) alternated irregularly (even from year to year, i.e. faster than habitat shifts that would have occurred in natural settings). Owing to their long lifespan^28^, grebes should have the potential to learn from past experience about the real value of novel habitats. I explored grebes’ settlement decisions in terms of territory fidelity/infidelity and their appropriateness (settling in good/poor habitat) in relation to birds’ previous-year breeding experience and the previous- and current- year habitat quality of former and new territories. In particular, I focused on information gathering and decision making by breeders from low-quality territories, as I expected territory movements to be more frequent among birds trapped in ill-suited habitat. I also predicted that quick alternation of fish stocks disrupting temporal covariation of habitat quality in ponds would render grebes’ settlement decisions vulnerable to bias.

## Methods

### Study system and the mechanism of the ecological trap

The study was conducted on semi-natural ponds used for extensive common carp (*Cyprinus carpio*) farming in eastern Poland (50.90–51.45′ N, 21.97–22.43′ E; see^28^ for a map of the study area). Natural ponds and wetlands were virtually absent in the study area due to drainage or conversion to aquaculture. The ponds predominantly used by grebes were stocked either with carp fry or with larger-sized, year-old fish. In ponds stocked with fry (hereafter: high-quality habitat), fish larvae, introduced in spring, were initially too small (approx. 3 mm individual total length) for consumption by grebes, but constituted ample food supplies later, during the chick-rearing stage (late May‒July; typical individual length at the end of June 60–70 mm^25^), together with rapidly increasing macroinvertebrate and amphibian resources. In contrast, in ponds with older/larger fish, stocked at lengths of approximately 110–160 mm (low-quality habitat), a fraction of smaller fish of a suitable size for adult grebes created attractive early-season food supplies. However, later in the season the fish exceeded the size usable as prey for chicks and negatively affected other prey resources of grebes, resulting in poor reproductive success. As prey availability at the time of territory establishment by grebes did not predict prey availability during the brood period, these ponds functioned as an ecological trap, with a large fraction of broods ending in failure (see^25,26^ for details on the study area). As grebes typically do not move broods among ponds, settling decisions of breeding birds are crucial for their reproductive success. Nest losses (associated mainly with egg predation and human disturbance, such as removal of reed beds) did not differ between high- and low-quality habitats; predation on breeding birds was infrequent^26^. Although the structurally similar ponds varied in some important habitat characteristics, such as size (area) or the amount of emergent vegetation (potentially increasing safety from disturbance and predators), fish size and density were roughly uniform among ponds stocked with the same age-cohorts. The fish stocking/removal regime was clearly decisive for food conditions for grebes, and thus provided an independent dichotomous criterion of the habitat quality of distinct habitat patches^26^. The ratio of available high-quality ponds to ponds providing low-quality habitat in the study area was variable between years, but on average roughly equal (1.1 ± SE 0.10). Fish age cohorts were alternated irregularly (from year to year—or stocks remained the same during more than one year) by stocking/removal in the drainable ponds. Thus, the same pond could function as a highly suitable habitat in one year and an inferior habitat in the next. The proportion of ponds where the age-stocks changed from year to year varied between sites and years, ranging from 0.2 to 1. The available habitats were not saturated; each year some low- and high-quality ponds that had been occupied in other study years were vacant, indicating little density-dependent regulation of habitat selection, i.e., undefended territories were not restricted to unfavourable habitat (cf^29^). Since on average clutches were initiated earlier on low-quality ponds than on high-quality ponds, breeders on low-quality ponds were unlikely to be subdominant individuals, pushed to inferior habitats^26^ (see also^30^).

### Red‐necked grebe settlement decisions and reproductive success

Data on pond occupancy, hatching and fledging success of grebes, and the habitat quality of individual ponds in each year were collected every year from 1996 to 2014 from about 50–70 ponds aggregated in clustered mosaics of low- and high-quality ponds. The nearest neighbouring ponds were often separated only by grassed levees, but clusters were situated at least 10 km apart. Data on fish stocking (habitat quality) were obtained from local fisheries. Grebes were ringed with a unique combination of a numeric metal ring and coloured plastic rings after capture in submerged nets or by night lighting^31^. The grebes used were marked adults, at least in their third calendar year (for ageing criteria see^24^), which nested (in year t) and then returned to breed in the study area the following year (t + 1). Most of them were ringed as adults, so their exact age was unknown. In the majority of pairs, only one member was ringed, but observations of pairs where both mates were known indicated high pair fidelity in the population; many breeders arrived at the ponds as established pairs (J. Kloskowski, unpublished data). Colour rings, visible above the surface during many of the birds’ activities, could be identified from a distance using a 60 × spotting scope. The recorded territory shifts ranged from movements between adjacent ponds, separated by levees, to 26 km. As pond habitat quality was determined by fish stocking, breeding pond was considered equivalent to territory (although larger ponds were used by more than one pair); i.e., territory shift was defined as a shift to a new pond, and not any territory movements within the same pond. Some breeders were likely to emigrate (temporarily) from the study area; however, breeding birds typically showed fidelity to the site (pond cluster). The average annual apparent survival of adult birds was about 79%; the population decreased during the study period, but very low recruitment, which may be indicative of large-scale declines, has been shown to be more responsible for the population trend than the use of low-quality habitat^28^. Birds were considered breeders if they at least initiated a clutch or if a nest was built and the pair was present in the territory during most of the season. Brood size was checked until both parents terminated parental care and left the territory, typically 6–8 weeks post-hatching^32^. Chicks that survived to this age were considered fledglings. Birds that had failed to hatch any eggs in the preceding year were included provided that they had stayed in the territory for most of the season (immediate leavers could not gather information about the pond’s food resources for young). Also, only pairs whose previous year’s breeding ponds were available in the subsequent year (filled prior to 1 May) were included. I did not attempt to assess potential improvement in habitat selection with age/breeding experience because the breeding histories of the returning long-lived adults were mostly incomplete. The effective sample size was 93 between-year fidelity and settlement decisions by 53 individually marked birds (pairs). Apart from routine field visits, continuous 1–8 h observations of territorial birds (altogether about 920 h) were made at least weekly in a subset of grebe pairs (see^26^ for more details on field methods), and visits of marked breeding or post-breeding individuals to other ponds were recorded. Some of the birds breeding on low-quality ponds made short-distance foraging trips to neighbouring ponds, although the flights became more frequent only during later brood-rearing stages and thus did not help to decrease chick mortality, which peaked during the early post-hatching period^25^.

### Statistical analyses

Generalized linear mixed effects models (GLMM) were used throughout. To account for repeated observations of the same birds (pairs), their identity was included as a random factor. Data were pooled between years. To investigate between-year stay-switch decisions and their appropriateness (settling in high-low habitat in year t + 1; treated as a binary variable), models with a log link and binomial distribution were performed. To include complete previous- and current-year habitat information in the analyses, full models regarding stay-switch decisions should principally contain variables on the quality of the previous-year and next-year territory in both years t and t + 1. However, in contrast to the breeders from low-quality ponds, most of those from high-quality ponds exhibited between-year pond fidelity (see Results), and thus variables representing pond quality in the models showed high multicollinearity. Therefore, first a ‘total’ model, based on the complete sample of breeding pairs, was constructed to compare breeders from high- and low-quality ponds (year t). To test the effect of the previous year's breeding success on the propensity to shift territories, hatching success in year t (complete clutch failure vs hatching of at least one egg) was included (Table 1). The previous year's hatch and not fledging success was used, as the latter was strongly correlated with habitat quality^26^, and it is clutch loss, especially due to predation, that often leads to territory change^17,33,34^. Since the probability of moving to a new pond differed between breeders from high- and low-quality ponds (see Results), they were subsequently analysed separately in two different models. In the GLMM including only breeders from low‐quality habitat, the main explanatory variables were habitat quality of the old breeding pond (occupied in year t) in year t + 1 and habitat quality in year t and in year t + 1 of the pond occupied in the subsequent year (Table 1). As all habitat variables were strictly correlated in the model on the breeders from high-quality ponds, it contained only one (the most varying) explanatory factor: the quality of the pond occupied in year t + 1. Along similar lines, since the quality of the old territory (year t) and the stay-switch decision were correlated, in the GLMMs examining factors affecting appropriateness of between-year settlement choices, breeders from high- and low-quality ponds were compared first; the other fixed factor was between-year habitat consistency of the pond occupied in year t + 1 (habitat quality consistent or changed from year t to year t + 1). Then, separate models for breeders from the two habitats tested whether the appropriateness of habitat selection in year t + 1 was related to the stay-switch decision.

**Table 1:** Results of GLMMs of the probability of shifting territory between years and making appropriate habitat selections (models with binomial errors and logit link function), and the probability of between-year change in breeding success (model with normal errors and identity link) of red-necked grebes nesting on fish ponds of contrasting habitat quality.

Parameter	Predictor	Estimate	Wald (df = 1)	n	*p*
**Territory shift**					
‘Total’ model	Quality of previous year's territory in year t (high, low)	0.00, 2.79 (0.58)	22.82	93	< 0.001
	Previous hatch (yes, no)	0.00, − 0.13 (0.65)	0.04		0.841
Breeders on low-quality ponds in year t	Quality of previous year's territory in year t + 1 (high, low)	0.00, 0.16 (0.93)	0.03	40	0.861
	Quality of next year's territory in year t (high, low)	0.00, − 3.61 (0.98)	13.56		< 0.001
	Quality of next year's territory in year t + 1 (high, low)	0.00, 1.11 (0.96)	1.32		0.259
Breeders on high-quality ponds in year t	Quality of next year's territory in year t + 1 (high, low)	0.00, 0.48 (0.79)	0.37	53	0.544
**Mistaken habitat decision in year t + 1**					
‘Total’ model	Quality of previous year's territory in year t (high, low)	0.00, 2.11 (0.66)	10.35	93	0.002
	Between-year consistency in quality of next year's territory (consistent, changed)	0.00, 2.84 (0.74)	14.57		< 0.001
Breeders on low-quality ponds in year t	Territory shift decision (stayers, switchers)	0.00, 1.59 (1.05)	2.28	40	0.131
Breeders on high-quality ponds in year t	Territory shift decision (stayers, switchers)	0.00, 0.41 (0.83)	0.25	53	0.617
**Change in breeding success between year t and year t + 1**	Quality of previous year's territory in year t (high, low)	0.00, 0.52 (0.47)	1.22	62	0.274
	Territory shift decision (stayers, switchers)	0.00, − 0.45 (0.47)	0.91		0.345

The models incorporate bird (pair) identity as a random effect. Non-significant interaction terms (*p* > 0.1) were excluded. Standard errors of effect estimates are given in brackets; for categorical factors, standard errors of differences are presented. Sample sizes for each model are reported. Settling on low-quality ponds was considered a mistaken habitat decision.

To assess whether breeders from low- and high-quality habitat improved their reproductive success in year t + 1 depending on their territory quality in year t and the stay-switch decision, the between-year difference in fledgling production was analysed using a normally distributed GLMM with an identity link function. Pairs that failed to hatch young in year t + 1 were omitted, as frequencies of nest failure (most of them caused by humans) were not related to habitat quality defined by food availability^26^.

Owing to the small number of variables examined (some models were univariate), inference was based on models including all predictor variables; non-significant interactions were dropped to improve model fit. Minimal models obtained using a backward stepwise procedure produced the same conclusions. All GLMMs were fitted using Schall’s technique^35^ in Genstat v. 15.

## Results

**Figure 1:**
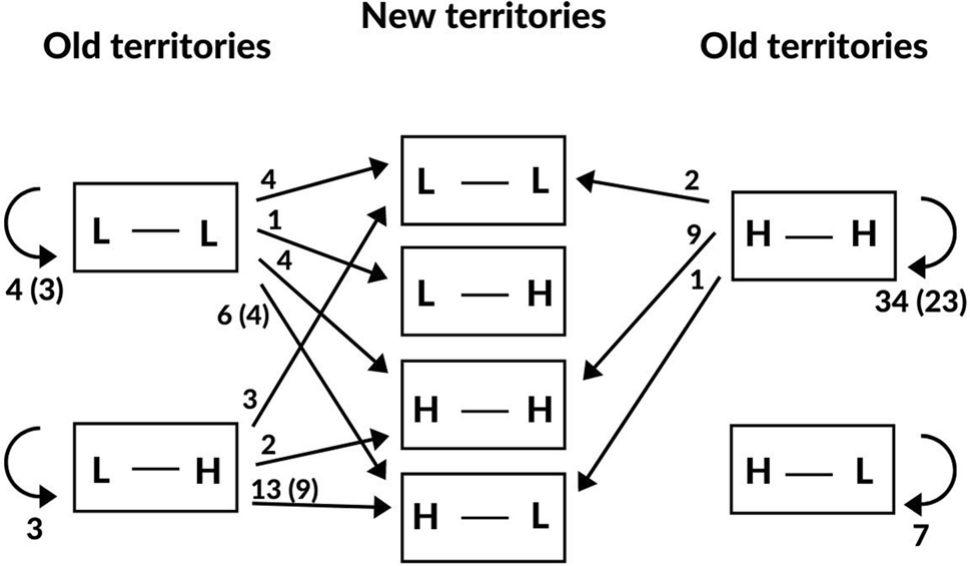
Between-year settlement decisions of red-necked grebes nesting on fish ponds of contrasting habitat quality. Combinations of letters within boxes indicate changes or consistency in habitat quality from year t to year t + 1, L—low-quality habitat, H—high-quality habitat. Old territories are ponds occupied in year t, new territories are ponds where birds shifted in year t + 1. Arrows indicate directions of between-year movements. Returning arrows represent pond fidelity between years. Numbers refer to sample sizes; numbers in parentheses were added when the same individuals were sampled more than once.

Birds breeding on low-quality ponds showed a greater propensity to move to a new breeding pond (33 territory shifts out of 40 year t/t + 1 dyads) than those breeding on high-quality ponds (12 of 53 dyads) (Fig. 1, Table 1). The tendency to change territory was not related to hatching success in the previous year (Table 1). Shifts from low-quality ponds could not, as a rule, have been forced by other grebes, because most of the abandoned breeding ponds (27 out of 33) remained vacant in year t + 1. In contrast, some of the birds moving away from high-quality ponds might have been evicted to less-preferred areas, because their former territories were usually occupied in the subsequent year (11 out of 12). However, occupancy by new pairs was a poor predictor of involuntary territory shifts by prior occupants; in year t, 20 of 53 analysed breeding attempts in high-quality habitat and 19 of 40 in low-quality habitat were on ponds shared with other pairs. The between-year stay-switch decisions by breeders from high-quality ponds were not related to the quality of the pond selected for breeding in year t + 1 (Table 1). Breeders from low-quality ponds were more likely to move to ponds that had been high-quality habitats in the previous year (25 out of 33 new ponds, Fig. 1) while the quality of the old and new territory in year t + 1 was not significant (Table 1). The switchers were not likely to base their decisions on social information obtained from conspecifics, because only nine (27%) of the new breeding ponds were occupied in the preceding year.

Data on prospecting were difficult to collect if the visited ponds were occupied, as the residents defended their territories vigorously, often forcing intruders to leave the pond. Altogether 16 breeders from low-quality ponds were observed to visit other ponds during the chick stage or immediately after breeding in year t; 13 (81%) established a breeding territory on the pond prospected a year ahead, and three (19%) settled on other ponds than those explored in the previous year (in one case the prospected pond was dry in the next year). The territorial and prospected ponds were typically within the same pond cluster, at a distance of up to 1 km. One territory shift, however, involved prospecting of another pond cluster at a distance of 12 km and dispersal to it the next year.

The probability of bad settlement decisions was higher when the habitat quality of the pond selected for breeding in year t + 1 was inconsistent between years t and t + 1. Breeders from low‐quality habitat occupied low-quality ponds in year t + 1 more frequently than breeders from high-quality ponds. The appropriateness of settlement decisions, analysed in separate models for the two habitats, was not related to territory (in)fidelity (Table 1). However, the observed pattern of better decisions by breeders from high-quality ponds could likely be biased by inconsistent alternation of fish stocks among ponds and years (Fig. 1). In most of the 53 year t/t + 1 dyads, ponds that were of high quality in year t were of high quality in year t + 1 as well; ponds that were of high quality in year t shifted to low habitat quality in year t + 1 in only seven cases; residents of all these ponds returned to the territory and failed to produce fledglings in year t + 1. The frequency of faulty decisions (settling in low-quality habitat) by switchers from low-quality territories was high (26 of 33 territory shifts). Pairs that remained faithful to territorial ponds that were of low quality in year t were roughly equally likely to find themselves in high or low-quality habitat in year t + 1, and were not significantly better off than the switchers (Fig. 1, Table 1). Among breeders from high-quality ponds, birds that shifted territories between consecutive years did not differ from ‘stayers’ in the probability of occupying a low-quality pond in year t + 1 (Fig. 1, Table 1). Fish-stock alternation patterns do not explain the high rate of territory switching and of wrong decisions by birds from low-quality ponds; of the 40 ponds that were of low quality in year t, 21 were of high quality in year t + 1, but only three of them were occupied by returning residents).

The change in fledging success between year t and t + 1 was independent of whether grebes had nested on high-quality ponds or on low-quality ponds in year t; predicted mean differences between years 0.14 and 0.66, respectively, standard error of differences 0.47 (Table 1). Similarly, territory switchers did not fare better than birds showing territory fidelity; mean differences 0.168 and 0.63, respectively, SED 0.47 (Table 1).

## Discussion

Red-necked grebes responded to the quality of their territory resources using a simple rule for territorial stay-switch decisions: to remain faithful to a high-quality pond from one year to the next and to abandon a low-quality pond after a poor season. These findings are conservative in that, in contrast to breeders on high-quality ponds, the shift from low-quality territories by most territory switchers was obviously voluntarily, i.e., they could not have been displaced by intruders^36^, apparently according to the rule that territories that were of low quality in the previous year were unattractive for a take-over (see below). The breeding outcome was highly related to habitat quality^26^, so it cannot be determined what cues on breeding conditions (e.g. pre-shift reproductive output or foraging performance) were decisive for subsequent territory shifts^18,33^. A possible cue could be foraging ease associated with the presence of fish of size suitable for consumption, since grebes preferred ponds with such fish during the brood stage when visiting other ponds to provision the chicks. Recent hatching success appeared to be of minor importance, presumably because predatory pressure on incubating birds, which could be crucial for territory change (cf^34^), was low^26^. However, some longer-distance dispersal could have biased the results, as pre‐hatching nest failure was occasionally followed by the immediate disappearance of the resident pair, which could re-attempt breeding in locations outside the study area.

Most of the breeders from low-quality habitat shifted between years to ponds that were of high quality in the year prior to the movement, irrespective of the current habitat quality of the new pond and of the former nesting pond (cf^37^). Undoubtedly, the territory selection process by grebes must rely to a significant degree on proximate cues available in the period between arrival and territory establishment, simply due to possible between-year environmental alterations of potential breeding habitats, such as water level fluctuations. However, the conjecture that information acquired during the previous breeding season was of significant relevance to the next year's decision-making on habitat choice is supported by the observations of many of the switchers visiting the future breeding pond during the brood stage or immediately after breeding. Previous-year information was somehow inherent in the decisions of birds breeding on high-quality ponds in year t, as most of them remained on the same territory in the following year. Multiple information sources, including social information about a habitat’s quality (presence of successfully breeding conspecifics or number of young per brood) might have been integrated by grebes^38,39^; however, switchers from low-quality territories apparently relied primarily on their own environmental sampling^16,40^.

Breeders from neither habitat type increased reproductive success in the next breeding season. This was not surprising in birds breeding on high-quality ponds in year t, but noteworthy in breeders from low-quality ponds, i.e. those that had acquired some experience with the unreliable habitat cues. They did not improve their habitat selection, although most of them attempted to correct their choices by shifting to other ponds. Breeders from high-quality ponds were less likely to make mistaken habitat decisions in the following year; however, the pattern observed was largely due to the inconsistency in the rotation of fish stocks by the local fisheries; some high-quality ponds were stocked with fry fish in more than one year in a row, so their returning residents selected high-quality habitat again in these years. These birds might have obeyed the same general rule (win-stay, lose-switch) as breeders on low-quality ponds, or simply ignored habitat cues (see also the ‘multiplier effect’^41^). When habitat conditions changed due to fish stocking in ponds previously providing good habitat, their residents that showed territory fidelity misjudged habitat quality as well. Thus, better settlement decisions of birds nesting on high-quality ponds did not necessarily mean that they had better cognitive abilities than breeders on low-quality ponds. On the other hand, conditions experienced in the past may influence an individual’s response thresholds to habitat alteration and its choosiness about prospective territories^6,10^. After breeding in a profitable habitat, birds need not necessarily stay in the same territory, but they may be responsive to cues similar to those from a habitat patch in which they have previously been successful. By contrast, experience limited to breeding in poor habitat patches may lead birds to be too willing to settle in another poor patch, analogously to the hypothesis of natal habitat preference induction^42,43^. It should also be remembered that since the exact age and entire breeding history of the marked birds was usually unknown, the present models refer to one-off choices, whereas settlement decisions are part of a sequence of choices made by individuals throughout their life history^15^ and influenced by experience from more than one breeding season. Again, the present results might be biased by an unknown fraction of breeders choosing to disperse to other locations. However, good-quality natural habitats, where the old behavioural rules might produce mostly positive fitness returns, were scarce in eastern Poland, and if the dispersers moved to other fish ponds, there is no strong reason to expect better settlement decisions beyond the study area than within it.

Why did most grebes breeding in poor habitat fail to select habitat more appropriately in the following year? Settling grebes were exposed to a double risk of error, as their decisions were based on habitat cues from the previous year and cues obtained early in the current season, which in both cases were time-limited. Prospecting for future breeding sites in one year and use of the information for dispersal and settlement decisions in the next is largely ubiquitous in migratory birds^21,36,37^. It is likely to be adaptive in grebes breeding in natural (ancestral) habitats; although these habitats may occasionally be subject to rapid changes typical of shallow waterbodies^24,44^, their food resources are obviously more consistent between years than resources of managed ponds, driven by cycles of fish stocking and removal. When the predictability of food for chicks is low at the time of habitat selection^1,30,45^, cues from the previous year collected during the brood stage may be a better predictor of habitat quality than cues gathered prior to breeding^37^. However, reliance on previous-year information may set animals up for failure in landscapes undergoing rapid changes (from year to year). While many species may learn to avoid perceptual pitfalls of current cues on habitat condition (sensu^46,47^), learning based on the previous-year assessment and memorization of the profitability of particular habitat patches (ponds) will be ineffective in the next year's selection of a breeding territory^2,38^. In grebes, learning about territory quality was apparently spatiotemporally constrained; breeding birds associated habitat quality with spatial location and did not recognize the temporal scale of environmental fluctuations. This would indicate that learning about habitat quality has evolved in this species as a response to spatial, rather than temporal, variation in autocorrelated environments^15,48^. The present data suggest that the rate of environmental change and its pattern (irreversible or recurring) are of no less importance for adaptive habitat selection than the relative availability of good and bad habitat patches. Here, the strength of the ecological trap set by the fish stocking practices was largely contingent on the proportion of ponds fluctuating between rich and poor conditions relative to ponds with consistent conditions between years (i.e., grebes, at least experienced breeders, could reliably learn about habitat patches that were consistent in quality).

Assessment of habitat quality could potentially be improved by more thorough sampling of potential territories (^14^), i.e. more frequent and longer-distance movements between habitat patches before, during, and after breeding. However, increased sampling entails enhanced energetic effort. Short foraging (and potentially prospecting) flights, in which a significant portion of the time is consumed in take-off, can be particularly costly due to high wing loading in grebes^24,49^. While species with low flexibility in prospecting for suitable habitat may be more strongly affected by HIREC, more frequent and larger-scale prospecting does not imply better habitat selection if settlement decision rules remain unchanged. A simple rule of thumb^50^ for grebes could be to associate the early-season presence of potentially competitive fish with food scarcity at the brood stage. In fact, grebes consistently avoided ponds with fish too large to be ingested by adult birds^26^, and a further step would be a generalization of the response from large fish to a broader range of fish sizes. Still, avoidance of potential territories with food resources abundant during the pre-breeding season (large yet available fish) requires ignoring a short-term rewarding stimulus, which can be a reliable cue in natural settings^14^. Learning to make path-independent^15^ settlement decisions, based exclusively on current-year cues, could be also hindered by the irregularity of the pattern of quality change in individual habitat patches (ponds). When birds considered individual ponds as potential territories in periods of habitat consistency in the ponds (the next year’s habitat conditions in the prospected ponds fit the previous-year information), the familiar cue-response system worked well and could be reinforced. The present data suggest that a wide array of ecological traps may be created by alternating management regimes in which animal resources undergo unpredictable, recurring shifts, such as crop rotations, as well as by rapid yet irregular climate changes producing a phenological mismatch between the breeding cycle and optimal environmental conditions^48,51^. An irregular pattern of resource change may disrupt the temporal covariation conditions relied on by animals learning to identify false habitat cues.

Informed territory choice (based on cues on whether and where to disperse), with a portion of individuals staying in high-quality patches while those from low-quality patches move away, has the potential to buffer abrupt environmental changes at the population scale^52^. However, successful decision making on breeding site (in)fidelity and settlement is contingent on the quality of the information obtained and how it is processed^19,53^. Thus, understanding of decision-making rules when the available habitat cues are decoupled from the expected fitness consequences^13^ is crucial for predictions of the ability of individuals and populations to cope with ecological traps. The present study reveals potential limitations, arising due to constraints on learning, to behavioural adjustment to HIREC traps, and may explain why animals commit errors despite some experience with novel circumstances. Grebes apparently used a hierarchical decision-making mechanism in their settlement choices^54,55^. At the lower level they were guided by cues on habitat quality encountered and perceived during the previous breeding season and on arrival in the following year; at a higher level they used a Darwinian algorithm, weighting the information obtained at different time periods and prioritizing the experience from the previous year (cf^56^). It is widely assumed that to disarm an ecological trap it is sufficient to reduce the discrepancy between the cues and the true quality of the habitat^4,13^. However, management to mitigate the effects of ecological traps, such as encouraging settlement away from poor habitat options^46,47^, can be more complex than simple manipulation of habitat cues. It must both track environmental change and match the temporal scale of animal decision‐making. Animal adjustment to dynamic, novel situations can be impeded by learning using outdated information-processing routines. The dominance of past experience in settlement decisions may impair the ability of species to keep pace with environmental changes, including climate changes, in a similar way to strategies over-relying on conspecific attraction or site fidelity^57^. To better deal with traps arising from temporal habitat variability, animals may need to both largely disregard past information^15,19,20^, as outdated because of HIREC, and modify the associations of early-season cues with later food availability in the territory. A release from cognitive and decision-making biases (here from overreliance on information obtained in the previous season) may be more difficult than learning to correctly value habitat cues, because cognitive algorithms organizing past experience and categorizing cues are hierarchically higher in decision-making processes than mere recognition of the cues.

## Supplementary Information

Supplementary Information.
