# Supplementary material for: Win-stay/lose-switch, prospecting-based settlement strategy may not be adaptive under rapid environmental change
Source: Sci Rep. 2021 Jan 12;11:570. doi: 10.1038/s41598-020-79942-3 (PMC7804401; doi:10.1038/s41598-020-79942-3)
Supplement: Supplementary file 1 — Supplementary Information. [file 41598_2020_79942_MOESM1_ESM.pdf]

Ghosts of learning past: win-stay/lose-switch, prospecting-based settlement strategy may not be adaptive under rapid environmental change

Janusz Kloskowski

Electronic Supplemental Material 1

Data on habitat quality of breeding ponds and on hatching and breeding success, and between-year habitat selection under high and low food conditions in red-necked grebes (*Podiceps grisegena*).

Pair ID (ring no.)	Previous year's territory, yr t	Previous year's territory, yr t+1	Hatching success, yr t	Fledgling number, yr t	Year t	Year t+1	Stay/switch decision	Next year's territory, yr t	Next year's territory, yr t+1	Hatching success, yr t+1	Fledgling number, yr t+1	Prospecting, yr t
SA 06055	high quality	high quality	success	2	1996	1997	stay	high quality	high quality	success	2	
SA 06057	high quality	high quality	success	4	1996	1997	switch	high quality	high quality	success	3	
SA 06060	high quality	high quality	success	3	1997	1998	stay	high quality	high quality	failure	0	
SA 06064	high quality	high quality	success	3	1998	1999	switch	low quality	low quality	success	1	
SA 10024	high quality	low quality	success	3	1999	2000	stay	high quality	low quality	failure	0	
SA 10013	high quality	high quality	success	4	1999	2000	stay	high quality	high quality	success	4	
SA 10033	high quality	high quality	success	3	2000	2001	stay	high quality	high quality	failure	0	
SA 17747	high quality	low quality	failure	0	2001	2002	stay	high quality	low quality	failure	0	
SA 17746	high quality	high quality	success	2	2001	2002	stay	high quality	high quality	success	2	
SA 17745	high quality	high quality	success	4	2001	2002	switch	low quality	low quality	failure	0	
SA 17738	high quality	high quality	success	1	2001	2002	switch	high quality	high quality	success	2	
SA 10037	high quality	high quality	failure	0	2001	2002	switch	high quality	high quality	success	2	
SA 10035	high quality	high quality	success	2	2001	2002	stay	high quality	high quality	success	2	
SA 10033	high quality	high quality	failure	0	2001	2002	stay	high quality	high quality	failure	0	
SA 10037	high quality	high quality	success	2	2002	2003	stay	high quality	high quality	success	2	
SA 10029	high quality	high quality	failure	0	2002	2003	stay	high quality	high quality	success	3	
SA 02580	high quality	high quality	success	2	2002	2003	stay	high quality	high quality	failure	0	
SA 02578	high quality	high quality	success	2	2002	2003	stay	high quality	high quality	success	1	
SA 10029	high quality	high quality	success	3	2003	2004	switch	high quality	high quality	failure	0	
SA 02597	high quality	high quality	failure	0	2003	2004	switch	high quality	low quality	success	2	
SA 02580	high quality	high quality	failure	0	2003	2004	stay	high quality	high quality	failure	0	
SA 02578	high quality	high quality	success	1	2003	2004	stay	high quality	high quality	success	1	
SA 01703	high quality	high quality	success	2	2003	2004	switch	high quality	high quality	failure	0	
SN 02651	high quality	high quality	success	2	2004	2005	switch	high quality	high quality	success	2	
SA 02594	high quality	high quality	success	3	2004	2005	stay	high quality	high quality	failure	0	

SA 02589	high quality	high quality	success	2	2004	2005	stay	high quality	high quality	success	2	
SA 02578	high quality	high quality	success	1	2004	2005	stay	high quality	high quality	success	2	
SA 01703	high quality	high quality	failure	0	2004	2005	stay	high quality	high quality	success	2	
SA 17739	high quality	high quality	success	2	2005	2006	switch	high quality	high quality	success	1	
SA 17733	high quality	high quality	success	2	2005	2006	switch	high quality	high quality	failure	0	
SN 02657	high quality	high quality	success	2	2005	2006	stay	high quality	high quality	success	3	
SN 02653	high quality	high quality	success	2	2005	2006	switch	high quality	high quality	failure	0	
SA 02589	high quality	low quality	success	2	2005	2006	stay	high quality	low quality	success	0	
SA 02588	high quality	high quality	success	2	2005	2006	stay	high quality	high quality	failure	0	
SA 17739	high quality	high quality	success	1	2006	2007	stay	high quality	high quality	success	1	
SN 02657	high quality	high quality	success	3	2006	2007	stay	high quality	high quality	success	1	
SA 02593	high quality	high quality	failure	0	2006	2007	stay	high quality	high quality	success	1	
SA 02588	high quality	high quality	failure	0	2006	2007	stay	high quality	high quality	success	2	
SA 17733	high quality	low quality	failure	0	2007	2008	stay	high quality	low quality	failure	0	
SN 02670	high quality	high quality	failure	0	2007	2008	stay	high quality	high quality	success	3	
SN 02659	high quality	high quality	failure	0	2007	2008	stay	high quality	high quality	failure	0	
SA 17733	high quality	high quality	failure	0	2009	2010	stay	high quality	high quality	success	2	
SN 01723	high quality	low quality	success	3	2009	2010	stay	high quality	low quality	failure	0	
SA 02588	high quality	high quality	success	3	2010	2011	stay	high quality	high quality	success	2	
SN 08114	high quality	high quality	success	1	2011	2012	stay	high quality	high quality	success	3	
SN 02697	high quality	low quality	success	3	2011	2012	stay	high quality	low quality	failure	0	
SN 02668	high quality	low quality	success	2	2011	2012	stay	high quality	low quality	success	0	
SA 02588	high quality	high quality	success	2	2011	2012	stay	high quality	high quality	failure	0	
SN 08119	high quality	high quality	success	3	2012	2013	stay	high quality	high quality	success	2	
SN 08114	high quality	high quality	success	3	2012	2013	stay	high quality	high quality	success	3	
SN 08126	high quality	high quality	success	2	2013	2014	stay	high quality	high quality	success	3	
SN 08114	high quality	high quality	success	3	2013	2014	stay	high quality	high quality	success	2	
SN 08126	high quality	high quality	success	3	2014	2015	stay	high quality	high quality	failure	0	
SA 06061	low quality	low quality	success	1	1997	1998	switch	high quality	low quality	success	1	
SA 06064	low quality	low quality	success	0	1997	1998	switch	low quality	high quality	success	3	future breeding pond
SA 10012	low quality	low quality	success	2	1997	1998	switch	low quality	low quality	failure	0	
SA 06061	low quality	high quality	success	1	1998	1999	switch	low quality	low quality	success	2	future breeding pond
SA 10021	low quality	low quality	success	0	1998	1999	switch	low quality	low quality	success	1	
SA 10024	low quality	high quality	success	1	1998	1999	stay	low quality	high quality	success	3	other pond(s)
SA 10025	low quality	low quality	success	1	1998	1999	switch	high quality	high quality	success	4	other pond(s)
SA 06061	low quality	low quality	success	2	1999	2000	switch	high quality	low quality	success	0	

SA 06064	low quality	low quality	success	1	1999	2000	switch	high quality	high quality	failure	0	
SA 10021	low quality	low quality	success	1	1999	2000	switch	high quality	low quality	failure	0	future breeding pond
SA 17741	low quality	high quality	success	3	2001	2002	switch	high quality	low quality	success	1	
SA 02579	low quality	high quality	success	2	2002	2003	switch	high quality	low quality	success	0	future breeding pond
SA 02581	low quality	low quality	failure	0	2002	2003	switch	high quality	high quality	success	2	
SA 17741	low quality	high quality	success	1	2002	2003	switch	high quality	low quality	success	2	
SA 17745	low quality	low quality	failure	0	2002	2003	switch	high quality	high quality	success	2	
SA 17750	low quality	low quality	success	0	2002	2003	stay	low quality	low quality	failure	0	
SA 02579	low quality	high quality	success	0	2003	2004	switch	high quality	low quality	success	1	
SA 02588	low quality	high quality	failure	0	2003	2004	switch	high quality	low quality	success	0	
SA 02589	low quality	high quality	success	0	2003	2004	stay	low quality	high quality	success	2	other pond(s)
SA 02600	low quality	high quality	success	1	2003	2004	switch	high quality	low quality	success	0	future breeding pond
SA 17741	low quality	high quality	success	2	2003	2004	switch	high quality	low quality	success	1	future breeding pond
SA 17747	low quality	low quality	failure	0	2003	2004	switch	high quality	low quality	success	0	
SA 17750	low quality	low quality	failure	0	2003	2004	switch	high quality	low quality	success	0	
SN 01709	low quality	low quality	success	1	2004	2005	switch	low quality	low quality	failure	0	future breeding pond
SN 01710	low quality	high quality	success	0	2004	2005	switch	high quality	low quality	success	2	future breeding pond
SN 01718	low quality	high quality	success	2	2004	2005	switch	high quality	low quality	success	0	future breeding pond
SA 02579	low quality	high quality	success	1	2004	2005	stay	low quality	high quality	success	2	
SA 10026	low quality	high quality	success	0	2004	2005	switch	high quality	low quality	failure	0	
SA 17741	low quality	high quality	success	1	2004	2005	switch	high quality	low quality	failure	0	future breeding pond
SN 01718	low quality	high quality	success	0	2005	2006	switch	low quality	low quality	failure	0	
SN 02663	low quality	high quality	success	2	2005	2006	switch	high quality	low quality	failure	0	future breeding pond
SA 17747	low quality	low quality	failure	0	2005	2006	switch	high quality	low quality	success	1	
SA 10035	low quality	high quality	success	0	2006	2007	switch	high quality	high quality	success	2	future breeding pond
SN 01711	low quality	high quality	success	1	2007	2008	switch	low quality	low quality	failure	0	
SN 02681	low quality	low quality	success	1	2007	2008	stay	low quality	low quality	success	1	
SN 02694	low quality	low quality	success	2	2007	2008	switch	low quality	low quality	success	1	future breeding pond
SN 02681	low quality	low quality	success	1	2008	2009	stay	low quality	low quality	success	2	
SA 17733	low quality	high quality	failure	0	2008	2009	switch	high quality	high quality	success	0	future breeding pond
SN 02683	low quality	high quality	failure	0	2009	2010	switch	high quality	low quality	success	1	
SN 02668	low quality	low quality	success	0	2012	2013	stay	low quality	low quality	failure	0	

Pair ID - if both pair members were ringed, the ID of the bird that was re-sighted in more years was used

Hatching success - a nest was considered successful if at least one egg successfully hatched
